# Barriers and facilitators to expanding the role of community health workers to include smoking cessation services in Vietnam: a qualitative analysis

**DOI:** 10.1186/s12913-014-0606-1

**Published:** 2014-11-26

**Authors:** Donna Shelley, Linh Nguyen, Hieu Pham, Nancy VanDevanter, Nam Nguyen

**Affiliations:** Department of Population Health, New York University School of Medicine, 227 East 30th Street, New York, NY 10016 USA; Institute of Social Medical Studies, Hanoi, Vietnam; Rush Medical College, Chicago, NY USA; New York University College of Nursing, Global Institute for Public Health, New York, NY USA

**Keywords:** Cessation, Health workers, Qualitative, Vietnam

## Abstract

**Background:**

Despite high smoking rates, cessation services are largely unavailable in Vietnam. This study explored attitudes and beliefs of community health workers (CHWs) towards expanding their role to include delivering tobacco use treatment (TUT), and potential barriers and facilitators associated with implementing a strategy in which health centers would refer patients to CHWs for cessation services.

**Methods:**

We conducted four focus groups with 29 CHWs recruited from four district community health centers (CHCs) in Hanoi, Vietnam.

**Results:**

Participants supported expanding their role saying that it fit well with their current responsibilities. They further endorsed the feasibility of serving as a referral resource for providers in local CHCs expressing the belief that CHWs were “more suitable than their clinical colleagues” to offer cessation assistance. The most frequently cited barrier to routinely offering cessation services was that despite enacting a National Tobacco Control Action plan, cessation is not one of the national prevention priorities. As a result, CHWs have not been “assigned” to help smokers quit by the Ministry of Health. Additional barriers included lack of training and time constraints.

**Conclusion:**

Focus groups suggest that implementing a systems-level intervention that allows providers to refer smokers to CHWs is a promising model for extending the treatment of tobacco use beyond primary care settings and increasing access to smoking cessation services in Vietnam. There is a need to test the cost-effectiveness of this and other strategies for implementing TUT guidelines to support and inform national tobacco control policies in Vietnam and other low-and middle-income countries.

**Electronic supplementary material:**

The online version of this article (doi:10.1186/s12913-014-0606-1) contains supplementary material, which is available to authorized users.

## Background

According to the 2010 Global Tobacco Survey, 47.4% of men in Vietnam are current smokers, a smoking prevalence that is the second highest among South East Asian countries (SEAC) [1]. If current smoking rates are not addressed it is estimated that in 10 years tobacco use will be responsible for about 25% of adult male deaths in Vietnam [2]. Promoting cessation is the key to reversing current global trends in tobacco-related mortality over the next few decades [2,3].

The World Health Organization’s (WHO) Framework Convention on Tobacco Control (FCTC) includes, as a core provision, increasing access to evidence-based tobacco use treatment [4,5]. Article 14 of the WHO FCTC states that “each country shall take effective measures to promote cessation and adequate treatment for tobacco dependence.” Despite the availability of evidence-based approaches to addressing tobacco use few low- and middle-income countries (LIMCs) have integrated Article 14 recommendations into the routine delivery of preventive services [5].

Barriers to implementing cessation services in public health systems in LMICs include inadequate training of health care providers and lack of systems and staffing to support these services [6,7]. In addition, although a growing literature demonstrates the effectiveness of strategies to increase the delivery of smoking cessation treatment in primary care, these studies have been conducted almost exclusively in high-income countries [8]. Effective implementation strategies include clinical reminder systems and linking health care settings with state and national telephone counseling programs via a referral system, thus offering providers the opportunity to delegate cessation counseling and follow-up [9-12].

Smoker’s quitlines are an effective population-based method for increasing the reach of evidence-based tobacco use treatment. However, in LMICs this may not be a feasible option [13]. In fact, the WHO recently published guidelines for implementing Article 14 which state: “In order to promote tobacco cessation and develop tobacco dependence treatment as rapidly as possible, and at as low a cost as possible, countries should use existing resources and infrastructure” [13]. In Vietnam, as in other LMICs, there are infrastructure elements, including a robust public health delivery system with an extensive network of community health workers (CHWs) (referred to as village health workers in Vietnam) that could be leveraged to enhance tobacco use treatment. In LMICs, CHWs build bridges between the formal health system and communities increasing access to health services [14,15]. CHWs are critically important members of the public health care system. Therefore, it is surprising that in LMICs there are no studies evaluating the role of CHWs and/or village health workers as a referral resource for increasing access to evidence-based smoking cessation services, and we are aware of only one study in the U.S [16].

Because in Vietnam this workforce is referred to as village health workers (VHWs) we will use that term in this article. The primary purpose of this study was to examine the attitudes and beliefs among VHWs towards expanding their role to include delivering smoking cessation interventions and potential barriers and facilitator associated with implementing a VHW-delivered cessation intervention.

## Methods

### Study subjects and recruitment

The Vietnamese health care system is organized into four administrative levels: central, province, district and commune level. At the central level is the Ministry of Health (MOH), which formulates and implements national health policies and programs. The provincial-level health system consists of Provincial Health Departments and Preventive Health Centers, which are administered by the Provincial People’s Committee in each province. At the district level, the District People’s Committee administers district health centers and district-level hospitals. Within districts the commune health centers (CHCs), which are comparable to community health centers in the US, serve as the primary access point for public health and preventive care services in Vietnam, each providing services for an average of 5000–7000 people in its surrounding community.

CHCs are staffed with five to six commune health providers including one physician and three to five other health professionals (e.g., assistant physicians, nurses, midwives and/or pharmacists). In addition, each CHC is supported by a network of eight to 10 VHWs who conduct outreach in the villages where their CHC is located. They are under the direct management and direction of the head of the CHC and, in addition to coordinating with the CHC medical staff, they collaborate with local leaders and community-based organizations in their village.

VHWs have five main responsibilities: 1) delivering health information; 2) delivering counseling on disease prevention; 3) promoting maternal and child health and family planning; 4) providing first aid care and referring patients to the CHC; and 5) implementing national health programs at the village level. In addition, they serve as a bridge between the local CHCs and the community by, for example, collaborating with clinicians at CHCs to provide home-based follow-up to ensure that patients are adhering to clinical care plans. VHWs work within a highly formal, vertical organizational structure in which they do not have the autonomy to develop new programs or adapt current prevention program to the local context.

### Participant recruitment

We recruited participants from four CHCs in Hanoi Province. Together, these CHCs have 89 VHWs. Potential participants were invited to a meeting by the Director of each of the CHCs during which the research assistant provided an overview of the study. VHWs who were interested in participating were asked to provide contact information and sign up for pre- specified dates and times for the focus groups. Participants were eligible if they were working for at least one year as a VHW at the time of recruitment, and each received $4 (80,000 VND) as compensation for his or her time. The institutional review boards of the Institute of Social Medical Studies (ISMS) and the New York University School of Medicine approved this research.

### Data collection

Four focus groups were conducted in November 2012 with seven to eight VHWs in each (n = 29). A doctoral level facilitator from ISMS, the partnering research institution in Vietnam, conducted the focus groups. We used a grounded theory approach in developing the interview guide (Additional file 1) that was informed by Bowen’s key areas of focus for assessing the feasibility of implementing evidence-based interventions or new care processes (e.g., acceptability, practicality) [17,18]. The guide was developed in English and then translated into Vietnamese by an ISMS translator who is both fluent in Vietnamese and English. The lead investigator in Vietnam, who is bilingual, reviewed the translation. The focus groups began with background questions about VHWs’ scope of work, challenges in carrying out their current responsibilities, and their knowledge, attitudes and beliefs about current national and local tobacco control activities, including cessation services. We then explored their attitudes towards expanding their role to include smoking cessation counseling, and the acceptibility and practicality of serving as a referral resource for their CHC colleagues. Finally, we explored the potential barriers and facilitators (e.g. policy, training) related to implementing a VHW-delivered cesstion program. Focus groups were digitally recorded and transcribed verbatim in Vietnamese by ISMS before translating the transcripts into English for analysis.

### Analysis

Content analysis was conducted by two members of the research team (DS, HP). ATLAS.ti, 6.1, a qualitative analysis software program, was used to facilitate the analysis, but the focus group coding was undertaken manually. Data analysis consisted of a three-level coding process: open coding of all four transcripts by both members of the research team to identify relevant patterns and preliminary codes in the interviews, followed by focused coding to identify clustered concepts and to organize ideas, and finally identification of major themes [19-21]. Regular discussions between DS and HP were held to achieve consensus on emerging themes from the descriptive to the analytical stages. A final codebook was developed and finalized during this iterative process. All transcripts were then independently coded by the same two research team members.

## Results

### Participant characteristics

Participants ranged in age from 33 to 67 (mean = 53, SD = 8). Thirty five percent of the 27 participants were male. The number of years as a VHW ranged from one to 43 (mean = 22.5, SD = 7.1) with 56% in the position for 20 or more years. Forty four percent of the male participants (n = 4) were current smokers. No female participants reported current tobacco use.

### Themes

The analysis revealed four major themes: knowledge and beliefs about Vietnam’s current tobacco control program, attitudes towards expanding VHWs’ role to include cessation services, challenges to implementing VHW-delivered cessation services, and suggestions for addressing barriers. The findings are organized by main themes with sub themes where relevant.

#### Theme 1: Knowledge and beliefs about Vietnam’s national tobacco control program

Knowledge and beliefs about recent changes in policies and programs: VHWs described recent efforts by the Vietnamese government to implement new tobacco control policies and programs that are consistent with the FCTC. Specific examples included recent smoking bans: “The ban of tobacco use appeared at every public area, including on the public transports.” “We also have the ban on smoking in the meetings and workshops.” Others described new warning labels and a large scale media campaign: “Information about tobacco cessation is disseminated everywhere”.

A few VHWs described examples of inconsistencies in enforcement of smoke-free air policies, “There were no tobacco use at the weddings, but I can see people smoking at the meetings and workshops this year.” However, most expressed the belief that the new policies were changing social norms and behaviors. For example, one participant described changes that have occurred at formal events: “Tobacco is not displayed and smoked officially at weddings, funerals, banquets and feasts. This proves that our community’s awareness has changed positively.” Another participant described changes in individual smoking behaviors as a result of knowledge of the risks of secondary smoke: “They are more aware of the dangers of smoking to other people. They would go outside to smoke or do not smoke at the public areas”.

Perceived gaps in national program: Despite an ongoing media campaign to educate the public about the harms of tobacco use, warning labels on cigarette boxes and smoke free air policies, there was consensus among VHWs that there were no programs to help smokers quit. As one VHW further explained, they have not been “assigned” to help smokers quit: “They [public health authorities] have not launched a particular program on tobacco cessation. There is no specific program for us to do.” Another participant described the lack of focus on cessation in the mass media messages: “The information broadcasted on mass media focuses on the dangers of tobacco only, not the methods of tobacco cessation.” Another noted that in the CHCs cessation was not seen as important as other health issues: “The tobacco issue has not received the attention from the medical staff compared to other issues such as nutrition or other diseases”.

Despite the lack of a formal smoking cessation program several VHWs described providing informal advice to community members who smoke. One explained her efforts: “Of course, we still encourage and give advice to smokers to quit if we meet them as it is our duty. We tell them what we know about the dangers of tobacco”.

#### Theme 2: VHW attitudes towards attitudes towards expanding VHWs’ role to include smoking cessation counseling

During the focus groups the facilitator described a potential new model of care for treating patients who use tobacco that would place VHWs in a central role as a referral resource for clinicians in the local CHCs. The model would include training the clinicians to offer smokers brief advice and to refer smokers, who were interested in additional assistance, to VHWs who would be trained to offer education, counseling and information about other community-based cessation services. The focus groups explored VHWs’ attitudes towards and acceptability of expanding their role to include smoking cessation and how this additional job responsibility would fit into their current activities.

VHWs uniformly endorsed the program concept, even suggesting that they were more “suitable than their clinical colleagues” to provide more intensive counseling and ongoing cessation assistance outside of the CHC setting: “It is more suitable and effective if we rather than other people working in other departments give counseling to smokers to quit.” The belief, among participants, that they were the most appropriate health care worker to offer cessation assistance was consistently linked to their belief that they have a unique relationship with community members. One VHW described the intimate knowledge of the population as an advantage when trying to engage people in preventive services: “We are a great fit to do this program as we are the people who know thoroughly the characteristics of the population at our localities.” Another VHW pointed out the deep trust and respect village members have for them: “We have close relationships with people and they trust us. They even ask us for information and advice much more than the health staff at the commune health center”.

Most VHWs described cessation counseling and education as consistent with their scope of work. One VHW said: “Working as VHWs, we have the responsibility to protect the public health. This program is not outside that goal.” Finally, most indicated confidence in expanding their role: “We have done successfully many other programs such as family planning or keeping sanitation…we have been trained on all of that. If we are trained, we can do this [smoking cessation]”.

#### Theme 3: Potential challenges to implementing VHW-delivered cessation services

VHWs described several challenges to implementing a VHW-delivered cessation program. The most commonly described barriers to routinely offering smoking cessation education and counseling were the current lack of a specific program defined by the Ministry of Health, and their lack of authority to implement cessation workshops or educational events without direction from public health authorities: “We are doing programs directed by senior departments. We do not decide by ourselves about what to do.” Another VHW expanded on this theme by describing a lack of policies to support VHW engagement in smoking cessation interventions: “There is no regulation issued by the government or program for us to help smokers to quit. There are no competitions or assessment criteria for such a program. For example, for the immunization program, we need to reach a certain percentage of people having vaccinations. There is no such assessment for the tobacco cessation”.

Another challenge is the lack of training: “The job requires us to have comprehensive knowledge and information so that we are able to give people the right information persuasively. We cannot give people effective counseling if we do not understand the issue thoroughly.”

Home visits are a central component to implementing prevention priorities. Therefore, as several VHWs indicated, adding this program to their present workload would present challenges because of the extra time needed to conduct after hours home visits with the mostly male smokers who work during the day: “We need to go to their houses outside working hours in order to meet them as they go to work. It takes time.” Finally, one VHW noted that cultural beliefs often present barriers to implementing prevention programs: “Even when we select the right people to implement our counseling, we may still fail to do so. It is because they (the client) apply their folk experience instead of our information and knowledge”.

#### Theme 4: Suggested strategies for addressing potential challenges

Need for support of the government authorities: VHWs provided several recommendations to address the challenges they described. They reiterated the need to have approval from public health authorities to implement cessation interventions. One VHW added that “support would need to go beyond that of the senior departments to include the involvement of staff in the local CHCs”.

Need for community collaborators: Several VHWs emphasized the need to gain support from different members of the community including community-based organizations and village leaders: “We need the support from departments and different sectors, first is the head of the village. One person alone cannot do anything”.

One participant provided specific examples of potential community partners: “For example, we may invite medical staff to talk about the dangers of tobacco use at the Women Union, Youth Union, Veterans’ organization, and the elderly association. We need the support from all of those organizations at the same time in disseminating information about tobacco cessation”.

Another participant noted the value of having smoking cessation messages reinforced by multiple stakeholder groups including employers: “VHWs alone cannot help smokers to quit. This needs the collaboration and support among many departments, organizations, and authorities. For example, we give smokers’ counseling to quit at their locality. They are also advised to do so at their working places. People at the party meeting tell them about the dangers of tobacco as well. By giving counseling to smokers many times at many places, we believe that we can help smokers to quit”.

Finally, one participant noted the importance of including family members to support smokers’ quit attempts: “We can only talk to smokers one or two times but their family members can talk to them more than that”.

Need for training: VHWs provided additional details on the type of training they believed would be helpful which included filling knowledge gaps regarding tobacco use and the need for communication skills. “In order to implement the program of helping smokers to quit, we need to be trained on both knowledge and skills.” Another participant agreed that VHWs needed training on how to communicate effectively: “We also need to have skills that we can approach to smokers more closely and effectively so that they would listen to us.” Although their current knowledge about smoking cessation is primarily based on their exposure to media campaigns, all of the VHWs expressed confidence that with training they could offer effective interventions: “If we are trained, we can help smokers to quit”.

Need for financial support: Several participants mentioned low salaries as one of the challenges of working as a VHW, particularly given their large scope of activities: “The job costs us a lot of time and effort. But we need to hold more than one job to guarantee our family’s expense.” It was in this context that participants raised the need for financial support as an important factor for enhancing adoption of a new cessation program, including additional salary support for VHWs: “it is good if VHWs can get better remuneration after training”.

Incentives for smokers to participate were also viewed as a key component of a successful program: “If we want to invite people to go for a meeting, we need to provide each of them at least 10,000VND; otherwise, they will not go for the next time”.

## Discussion

VHWs described their current roles and responsibilities as consistent with offering community- based smoking cessation assistance to CHC patients and village members. They also described what they perceive as particular advantages of expanding their role to include this preventive services compared to their CHC colleagues. These included a deeper knowledge of the social context of smoking than health care providers and the strong trust and respect that village members have for VHWs as advocates for health promotion and disease prevention. VHWs further endorsed the specific referral model we describe in which health care providers would refer smokers to VHWs who would serve as “clinician extenders” offering more intensive treatment outside of the clinic setting.

Although VHW’s attitudes toward treating tobacco use were positive, they noted several policy- and organization-level barriers to expanding their role. The most commonly cited was that cessation services have not been defined as one of the national prevention priorities. This has several implications including the subsequent lack of training and other resources, and a lack of additional funding and incentives that accompany implementation of these priority programs. Moreover, they repeatedly explained that they and their colleagues in the CHCs do not have the authority to conduct programs without specific directives from the District health Directors and MOH.

As study participants’ comments indicate, the gaps in tobacco cessation services in Vietnam are not the result of a lack of commitment to tobacco control. In 2004 the Vietnamese government ratified the FCTC, and in 2012 it enacted an ambitious National Tobacco Control Action Plan (Decision No. 1315/QD-TTg) for the Implementation of the FCTC. As described by VHWs, Vietnam is implementing that plan with comprehensive smoke-free policies and hard-hitting media campaigns that are raising awareness of the dangers of tobacco use and changing social norms. Notably, the Action Plan calls for integrating cessation services into national health and education programs and builds on the MOH 2011 Annual Review which stated that strengthening the national infrastructure to ensure access to evidence-based cessation services is one of the MOH’s highest priorities [22].

Given the documented commitment to the full range of the FCTC treaty, it is important to ask why, in Vietnam and other LMICs, implementation of Article 14 has lagged behind other components of the treaty. One barrier may be the perception that providing cessation assistance would require an extra level of resources, including system changes and workforce development, not needed to implement smoke-free air policies and media campaigns. Additionally, despite evidence that counseling alone can reduce tobacco use, the emphasis on pharmacotherapy as a core component of treating tobacco use and dependence may also reduce enthusiasm in countries with limited public health resources. Finally, gaps in cessation services may be due to a lack of demonstrable cost-effective models for integrating adequate treatment of tobacco use into routine clinical and preventive service delivery.

The model we proposed to VHWs in the focus groups is based on a growing literature that demonstrates the benefits of simplifying the provider’s role by offering the means and opportunity to delegate additional counseling, in this case to a VHW [9-12,23]. Similar to models in which smokers are referred by clinicians to national or statewide telephone quitlines, in the proposed model providers would ask about tobacco use, advise patients to quit, and refer them for additional treatment to a network of trained VHWs.

A recent study conducted in Malaysia also supports findings that embedding referral systems in primary care can increase adoption and reach of tobacco use treatment guidelines [6]. This study, conducted in diabetes clinics, found that providing a referral to stand alone cessation clinics motivated clinicians to routinely provide cessation advice. However, the Malaysian model of creating stand-alone cessation clinics is too expensive to disseminate widely and stands in contrast to recommendations from the WHO’s recently published guidelines for implementing Article 14 which state: “In order to promote tobacco cessation and develop tobacco dependence treatment as rapidly as possible and at as low a cost as possible, countries should use existing resources and infrastructure” [13].

Consistent with WHO Guidelines for implementing Article 14, a VHW referral model would leverage the existing workforce and offer a potentially sustainable resource for ensuring wide access to treatment for smokers who want to quit. In Vietnam, as in other LMICs, community and village health workers have a strong track record of effectively delivering preventive services and increasing the reach of these programs [24-27]. They are also respected members of their communities, thus their involvement could contribute to changes in community norms regarding tobacco use and long terms sustainability of tobacco control efforts [24]. Notably, health care providers appear to support a VHW role in treating tobacco use. A survey of health care providers in 23 CHCs in Hanoi found that 90% strongly agreed that they would refer patients to a trained VHW for smoking cessation treatment, and 84% strongly agreed that VHWs could be trained to provide counseling [28].

Other challenges to expanding VHWs’ roles to include smoking cessation counseling were noted by focus group participants. These included a need for training that would both expand content knowledge and communication skills, an already heavy work load with salaries that do not necessarily match the time commitment required, and a need to engage other health care providers, community organizations and village leadership in any new smoking cessation initiative. However, next to training, the most commonly described challenge was the lack of a national program to integrate tobacco use treatment into the delivery of preventive services. According to focus group participants, explicitly making this a priority prevention program would address many of the other barriers.

This study has several limitations. First, the study was conducted with VHWs from urban CHCs. We therefore cannot be sure that the findings are generalizable to more rural settings. However, the scope of the work that the VHWs described, which is consistent, from their perspective, with offering cessation assistance, is standardized nationwide. Given the hierarchical nature of the public health care delivery system, the barriers described would likely apply to settings in other geographic location, but this requires additional confirmation. Second, the transcripts were translated into and coded in English. However, all translations were conducted by a bilingual RA and then reviewed by a second bilingual researcher (Dr. Nguyen). We believe this approach adequately addressed concerns about ensuring “conceptual equivalence” [29,30].

Our findings suggest that a team approach to treating tobacco use that allows providers to refer smokers to VHWs for further assistance is a promising model for extending the treatment of tobacco use beyond primary care settings and increasing access to evidence-based care. There is an urgent need to test the effectiveness and cost-effectiveness of this and other strategies for implementing tobacco use treatment guidelines in order to support and inform national tobacco control policies in Vietnam and other LMICs.

## Conclusions

Tobacco use continues to be the leading global cause of preventable deaths related to non-communicable diseases. Most of these deaths occur in LMICs, and this disparity is expected to widen further over the next several decades. Of the world’s 1.25 billion adult smokers, 10% reside within South East Asian Countries [31]. Therefore, it is critical to develop cost-effective strategies for promoting and disseminating guidelines for treating tobacco use that are adapted to the national context and priorities and leverage existing resources and infrastructure.

## Additional file

Additional file 1:
**Village health worker focus groups – moderator’s guide.**
