# Supplementary material for: Barriers and facilitators to expanding the role of community health workers to include smoking cessation services in Vietnam: a qualitative analysis
Source: BMC Health Serv Res. 2014 Nov 26;14:606. doi: 10.1186/s12913-014-0606-1 (PMC4247125; doi:10.1186/s12913-014-0606-1)
Supplement: Additional file 1: — Village health worker focus groups – moderator’s guide. [file 12913_2014_606_MOESM1_ESM.docx]

**Village Health Worker Focus Groups – Moderator’s Guide**

*[Notes in these parentheses are for the moderator – not to be read out loud]*

**Introduction**

Hello. Thank you for coming.

My name is ___________*[first name]* and I will lead the group discussion today.

You have been asked here today because of your experience as village health workers. For the next hour you will be part of a focus group. A focus group is when a group of people comes together to talk about a specific topic or to look at something and give their opinions and ideas. In a focus group people are brought together to think out loud about the topic and share their honest opinions.

As the moderator, I get to ask the questions, but I am not an expert in the topic. I am simply leading the group for the researchers.

OK. In today’s group we will be talking about helping smokers quit. As a *village health worker* you get to see and interact with patients or family members of patients who smoke. Therefore you may have insights and opinions that doctors don’t have. As you know, smoking is a serious health risk and so we look forward to hearing your thoughts about how we might reach and encourage smokers to quit. **As we discussed in the information sheet, we are not asking you to disclose any personal information and you don’t have to speak up and say anything if you choose not to**.

Usually when we get into the conversations, lots of ideas get brought up, and people can get quite involved in the discussion. So the only rule is, give everyone his or her turn to speak. I won’t be taking very many notes. I want to listen and talk with you. Therefore I’m going to tape record this session. I can then go over it later and remember what people said.

This research will be confidential. Everything we will discuss here is confidential. This means that your name will not appear in any report.

At the end of the interview/session, please fill out this form so that you can get paid in cash at the end of tonight’s focus group.

This meeting is going to last approximately 1 hour. May I begin?

**Introduction**

- I’d like us to go around the room and have everyone tell us your first name and how long you have worked as a village health worker.

**I. Roles and responsibilities as village health worker**

- What are your basic responsibilities as health worker?
- Everyone finds some parts of their job satisfying and rewarding, and everyone has some things they don’t fine satisfying or rewarding. For now, let’s focus on the things you **like** and find satisfying and rewarding about your role as village health worker. I’d like you to focus specifically on your interactions with the patients.

Probe - Eg., could you tell me more ….

OK. Thank you.

- And what about the interactions or roles with patients that you don’t like? Can you tell us about some of those situations?

Ok. Thank you

**II. Smoking Cessation**

Smoking is a common behavior in Vietnam, especially among men. We are trying to develop a program to help smokers quit. There are many effective treatments including counseling, but few smokers in Vietnam are receiving those treatments.

- How much of a problem do you think smoking is?
- How do you think smoking affects someone’s health? Probe: What about the impact on their family’s health?
- How does the importance of smoking compare to other health problems that you work on in terms of how much attention it gets from doctors and health workers?
- Why do you think so little attention is paid to this smoking cessation?
- What role do you think VHWs can play in helping smokers quit?

**III. Response to proposed new VHW role**

Now I’d like to hear your ideas about a specific role for VHWs that is focused on helping smokers quit. Some of you may be doing something like what I’m about to describe.

We are thinking about starting a program that would recruit VHWs and train them to offer brief counseling to help smokers quit. We know from research that it is easier for smokers to stop smoking when they have support from a counselor or health care provider.

Our idea is to train VHWs so that they are comfortable speaking to smokers in a way that may encourage them to quit. We would like to create a system in the commune health centers that would allow doctors and nurses to refer smokers who are interested in quitting to a trained VHW. The VHWs would be asked to meet at least twice with patients who agree to be referred to them by their commune health center doctor or nurse. Meetings could take place in person or by telephone.

Before I ask you what you think about this idea do you have any questions about what I just described?

- What are your reactions to this idea?

Probe: What do you like about the idea? What don’t you like?

- What do you think will be the difficulties or challenged in making this work?
- What do you think other VHWs like you would think about this new program?
- What would you need to take on this role?

Probe: what kind of training would you need?

- What ideas do you have to make this work?

How confident are you that you could carry out this role?”

Probe: Do you see yourself filling this role?

Does anyone have anything else that they’d like to say, perhaps something that we didn’t get to talk about.

*[If so pursue as time allows]*

*[If not, thank and dismiss]*

I would like to thank you all for your active participation in today’s discussion.
